# Asymptomatic Pneumopericardium with Atrial Fibrillation after Pericardiocentesis: A Case Report

**Published:** 2019-07

**Authors:** Hammad Shah, Momin Salahudin, Afrasyab Altaf

**Affiliations:** *Department of Cardiology, Rehman Medical Institute, Peshawar, Pakistan.*

**Keywords:** *Pericardial effusion*, *Pericardiocentesis*, *Pneumopericardium*, *Atrial fibrillation*

## Abstract

Air inside the pericardial cavity is called “pneumopericardium”, which is a rare complication of pericardiocentesis. Pneumopericardium may resolve spontaneously or can complicate into tension pericardium, requiring urgent aspiration. We herein describe a 55-year-old man with pericardial effusion who underwent pericardiocentesis. The patient was completely asymptomatic after the procedure. Chest radiograph and computed tomography scan accidentally detected pneumopericardium, which was subsequently complicated by atrial fibrillation and necessitated pharmacological cardioversion. We found no case of asymptomatic pneumopericardium complicated by atrial fibrillation after pericardiocentesis in our literature review. Clinicians and cardiologists should do a post pericardiocentesis chest X-ray to diagnose pneumopericardium and prevent the catastrophic complications of tension pneumopericardium.

## Introduction

Pneumopericardium is characterized by the presence of air around the heart chambers and inside the pericardial sac. It is more common in premature neonates with underdeveloped lungs due to vigorous resuscitation or assisted ventilation.^1^ In adults, it can be caused by blunt chest trauma^2^ and rarely it can develop following pericardiocentesis.^3^ It is very important to diagnose pneumopericardium because it can lead to a very dangerous consequence known as “tension pneumopericardium”, leading to hemodynamic compromise and needing urgent decompression.^4^

## Case Report

A 55-year-old man referred to our cardiology outpatient clinic with complaints of increasing shortness of breath, easy fatigability, malaise, and inability to lie flat for the last 15 days which had gradually worsened. The patient had also developed dry nonproductive coughs for the past 5 days, which woke him at night to sit upright. He complained of pressure symptoms while bending for prayers. This condition had affected his daily activities to the extent that he was unable to leave home. He had no past medical history of diabetes, hypertension, smoking, allergy, asthma, tuberculosis, chronic obstructive pulmonary disease, or heart failure. He had a good socioeconomic background. There was no history of recent travel.

On initial examinations, the patient was afebrile and had a regular low-volume pulse of 108 bpm, raised jugular venous pressure, blood pressure of 90/60 mmHg, and muffled heart sounds. The rest of the systemic examinations were unremarkable. A tentative diagnosis of pericardial effusion with cardiac tamponade was made. The patient was immediately admitted to the coronary care unit (CCU), where baseline investigations were commenced. Bedside echocardiography and 12-lead echocardiography were performed immediately, and they confirmed the diagnosis of massive pericardial effusion with features of cardiac tamponade. All the baseline investigations were within normal limits, and the patient had a high-sensitivity cardiac troponin level of 11.6 ng/dL. Immediate pericardiocentesis was planned, and informed written consent was obtained from the patient.

The patient was transferred to the catheterization laboratory. Under aseptic techniques and with 2% lidocaine as a local anesthetic, an incision was made at a left xiphocostal angle perpendicular to the skin and 3 to 4 mm below the left costal margin. After the confirmation of the position of the needle by echocardiography, a pigtail catheter was inserted via the Seldinger technique.^5^ Continuous electrocardiographic monitoring and vital recordings were maintained during the procedure. A total of 600 mL of fluid was drained straight during pericardiocentesis under colloid cover. He remained stable hemodynamically throughout the procedure, and his blood pressure improved to 110/70 mmHg after the drainage, which continued at 2 hour-intervals until he became dry, reaching a total of 780 mL. The catheter was clamped overnight. The next day, he improved subjectively and had a good night’s sleep. Echocardiography was performed for any recollection, and the result was negative.

The pericardial catheter was removed under aseptic techniques, and the patient was kept under observation. The pericardial fluid was sent for biochemical evaluations, which showed a fluid protein content of 5.5 g/dL and a total cell count of 1236 cells/cm^3^ with predominately 93% lymphocytes and 7% neutrophils. Gram-staining was negative, and Ziehl–Neelsen staining did not demonstrate acid-fast bacilli. The cytology report was negative for the presence of any atypical cells. Fluid GeneXpert was also negative. 

To confirm the diagnosis and ascertain the cause of the pericardium effusion, we planned a computed tomography (CT) scan of the patient’s chest with contrast. The CT scan was negative for any lung pathology; however, it surprisingly showed pneumopericardium causing mild right atrial collapse (Figure 1). 

**Figure:**
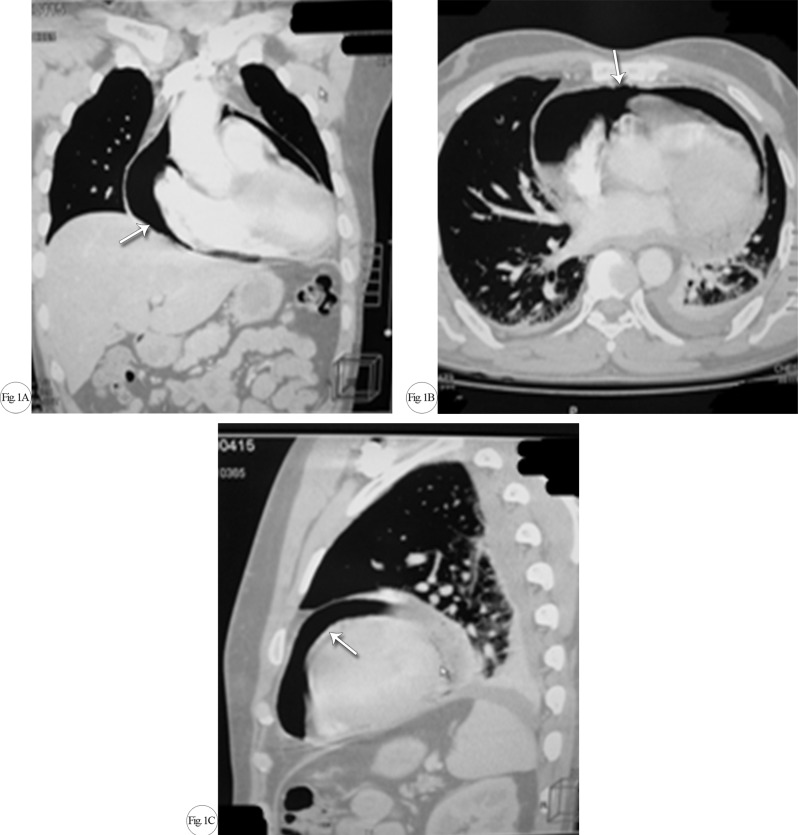


Given the CT scan findings, the patient was admitted to the CCU for observation with continuous cardiac and hemodynamic monitoring. Urgent bedside echocardiography was done, and it ruled out tension pneumopericardium. Chest X-ray was performed to evaluate the extent of the pneumopericardium and provide reference for follow-up (Figure 2). 

**Figure:**
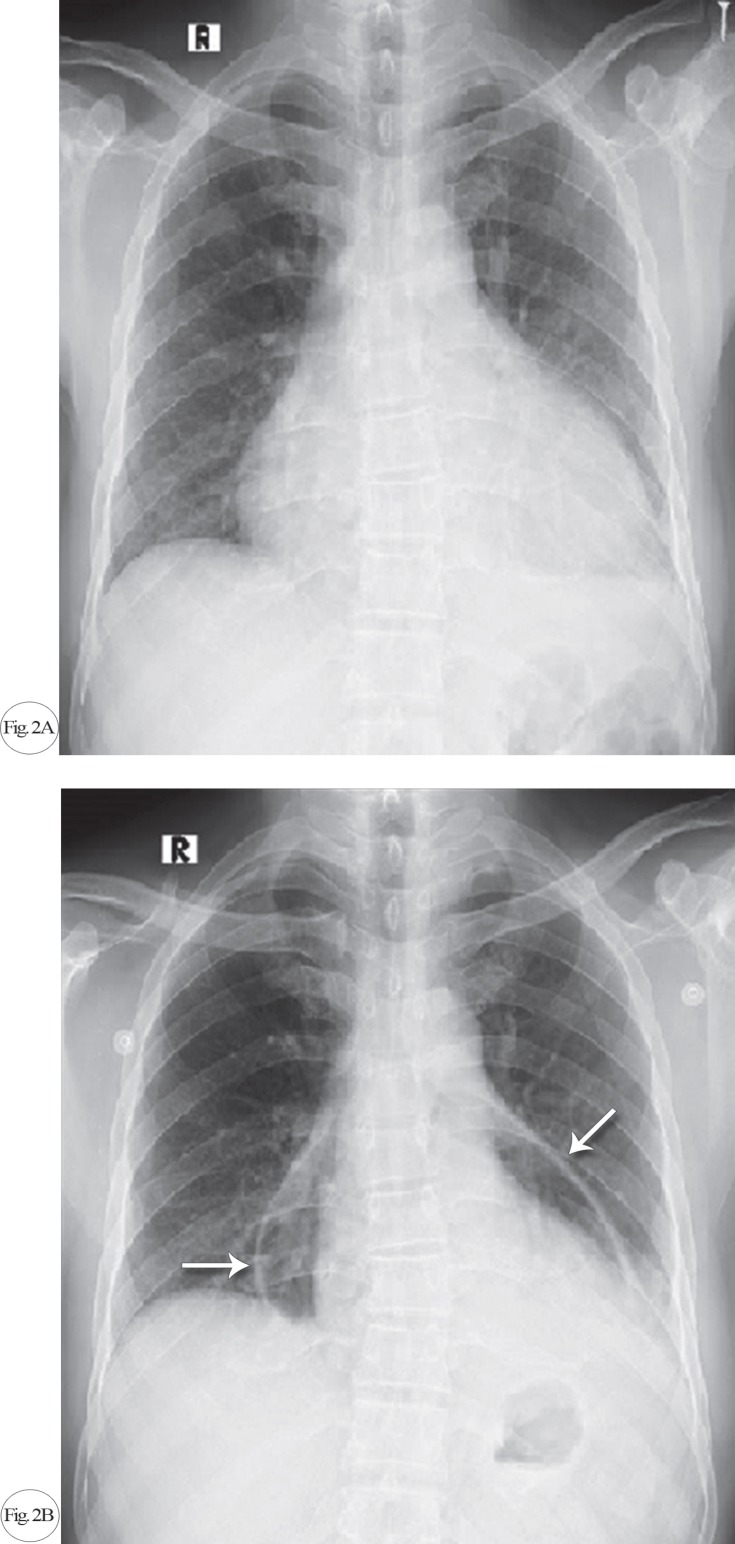


During his stay in the CCU, the patient developed atrial fibrillation, which was pharmacologically cardioverted after heparinization with enoxaparin and 300 mg of amiodarone administered intravenously stat in 100 mL of normal saline infused over 30 minutes. The sinus rhythm was maintained, and a maintenance dose of 600 mg of amiodarone was infused intravenously over 24 hours. After 48 hours of strict observation and vital monitoring, he was transferred to his room. Repeat chest X-ray was done after 72 hours, and it showed a reduction in the pneumopericardium. He made an uneventful recovery and was discharged home on empiric anti-tuberculosis therapy. A follow-up review after 2 weeks showed the complete resolution of the pneumopericardium with no pericardial recollection.

This case was unique insofar as no such example exists in which a case of pericardiocentesis is complicated by pneumopericardium and the patient remains asymptomatic despite developing atrial fibrillation.^2^^-^^4^ Informed written consent was obtained from the patient to use the clinical data, biochemical levels, and radiological images for academic and research purposes. The patient was satisfied with the management and appreciated the efforts made in his uneventful recovery.

## Discussion

Pericardiocentesis using catheter drainage under local anesthesia is the accepted treatment modality of choice to treat patients with pericardial effusion.^5^ The subxiphoid approach is the standard accepted approach to minimize complications.^6^ Pneumopericardium is a rare complication of pericardiocentesis occurring due to a leaky drainage system or due to direct pleuropericardial communication iatrogenically. It was previously discussed in a case report of a 20-year-old patient with tuberculous pericardial effusion.^7^ Chest X-ray film constitutes the initial diagnostic test for pneumopericardium and is characterized by a fine line of the pericardial sac outlining well a demarcated rim of air with the fluid level inside the cardiac silhouette.^8^ After pneumopericardium, the patient should be closely monitored for complications.^8^ Pneumopericardium, if there is no progression to tension pneumopericardium, may resolve spontaneously^9^ or may require pericardial aspiration.^9^ Our patient remained asymptomatic, hemodynamically stable, and was, thus, treated conservatively. 

## Conclusion

Clinicians and cardiologists worldwide need to be aware of the fact that pneumopericardium is a rare complication of pericardiocentesis and the patient can remain completely asymptomatic. We recommend that a chest X-ray be performed 24 hours after pericardiocentesis to exclude pneumopericardium. Although pneumopericardium resolves spontaneously most of the time, some cases can complicate into tension pneumopericardium, especially with comorbidities such as chronic obstructive pulmonary disease, which can be fatal.

## Notes:

***This paper should be cited as: ***Shah H, Salahudin M, Altaf A. Asymptomatic Pneumopericardium with Atrial Fibrillation after Pericardiocentesis: A Case Report. J Teh Univ Heart Ctr 2019;14(3):134-137.
